# Influence of Industrial Waste Gypsums in Excess-Sulfated Slag Cement: The Role of Wet Grinding

**DOI:** 10.3390/ma19050999

**Published:** 2026-03-05

**Authors:** Pei Tang, Hai Yang, Shuai Zhou

**Affiliations:** 1 State Key Laboratory of Silicate Materials for Architectures, Wuhan University of Technology, Wuhan 430070, China; 2 School of Materials Science and Engineering, Wuhan University of Technology, Wuhan 430070, China; hai-yang123@whut.edu.cn (H.Y.); zhous0525@163.com (S.Z.)

**Keywords:** industrial waste gypsums, wet grinding technology, excess-sulfated slag cement, hydration process

## Abstract

The rational utilization of industrial solid waste is an effective way to reduce environmental pollution. This study investigated the potential application of fluorogypsum (FG), flue gas desulfurization gypsum (FGD), phosphogypsum (PG), and titanium gypsum (TG) in the production of excess-sulfated slag cement (ESSC). It further investigated the effects of different types of gypsum on the performance and hydration process of ESSC through a wet grinding process. The results showed that as the pH value of the gypsum increased, the setting time of ESSC decreased, and hydration heat release occurred earlier. Phase analysis and microstructural characterization indicated that the type of gypsum affected the hydration rate, microstructure, and quantity of hydration products of ESSC, thereby influencing its compressive strength. To further improve the performance of ESSC, a wet grinding process was employed to enhance particle activity and promote hydration reactions. PG, due to its high solubility, demonstrated a better activation effect; after wet grinding, the 28 d compressive strength reached 40.03 MPa. Meanwhile, ESSC pastes prepared with high-pH FG exhibited not only good early strength (3-day strength of 21.93 MPa) after wet grinding but also excellent water resistance, with a softening coefficient of 0.96. This study clarifies the impact of gypsum type on ESSC performance and provides valuable insights for enhancing its properties.

## 1. Introduction

Finding a low-carbon, eco-friendly cement material to replace traditional cement in the construction industry has become a crucial strategy for achieving global sustainable development goals [1,2,3]. Utilizing industrial solid waste to produce building materials not only reduces energy consumption and environmental pollution during production but also promotes the efficient reuse of solid waste, thereby opening new avenues for the future development of sustainable building materials.

ESSC has attracted increasing attention from researchers [4]. ESSC is a typical low-carbon cementitious material with industrial solid waste as the main component. It is prepared by mixing industrial solid waste (accounting for more than 90% of the total mass), a small amount of alkali activator and appropriate admixtures, without a high-temperature calcination process. Its core components include gypsum (mass fraction > 40%), high-content blast furnace slag, and a small amount of alkali activator, which integrates the advantages of solid waste recycling, low energy consumption and low carbon emission. It is regarded as one of the key materials to promote the green transformation of the cement industry [5,6]. Compared to ordinary Portland cement (OPC), the raw materials of ESSC do not require high-temperature calcination, which fundamentally avoids the massive energy consumption and CO_2_ emission caused by the calcination of limestone in OPC production, reducing production costs by 30–40% and cutting CO_2_ emissions by approximately 70%. Its preparation method is straightforward and highly environmentally friendly [7]. In addition, ESSC has the characteristics of low hydration heat, which solves the problem of temperature cracks easily generated in large-volume concrete projects by OPC. Meanwhile, its late strength growth potential is better, partially compensating for its relatively low early strength [8].

However, long-term performance control of ESSC has always been a key technical difficulty restricting its large-scale application, especially the expansion problem caused by the delayed hydration of gypsum and slag. Although gypsum slag cement exhibits lower early compressive strength due to its slower initial hydration rate, its strength continues to increase as slag hydration progresses. With its low hydration heat, it is well-suited for large-volume construction projects [9,10,11]. To solve the expansion problem, it is necessary to optimize the type and dosage of gypsum, adjust the ratio of raw materials, and adopt appropriate activation technologies to control the hydration rate of the system, avoid the excessive generation of ettringite in the late hydration stage, and ensure the volume stability and long-term durability of ESSC. Therefore, gypsum slag cementitious materials merit extensive exploration and development for their environmental and economic benefits, playing a crucial role in promoting sustainable development in the cement industry.

Gypsum plays a pivotal role as a sulfate activator in the ESSC system. Compared to natural gypsum, which is limited in resources and costly to mine, industrial waste gypsums (IWGs) are widely used in research due to their abundant supply and low cost. This approach achieves the dual objectives of solid waste recycling and cost reduction [1,12,13,14]. Fei et al. [15] used FGD as a sulfate activator to activate slag; the sample exhibited excellent 28 d compressive strength and significantly improved mortar workability when the slag-to-gypsum ratio was 4:1 and the alkali activator content was 1.8%. Luo et al. [16] developed a composite cementitious material using FG that accommodates up to 60% FG content while exhibiting excellent mechanical properties and water resistance. Meng et al. [17] prepared green cementitious materials using modified PG, demonstrating that thermally activated and mixed ball-milled PG significantly enhances early strength, with increases reaching up to 174%. The maximum compressive strength and flexural strength attained were 40.1 MPa and 7.27 MPa, respectively. Ma et al. [18] investigated a composite cementitious system using blast furnace slag and FGD. At the optimal dosage, significant improvements were observed in mechanical properties, water resistance, and self-shrinkage performance.

However, existing research has primarily focused on the application of a single IWG, with a notable lack of systematic comparative studies on the activation characteristics of gypsum from different sources in ESSC. Although the main component of various IWGs is calcium sulfate, differences in production processes and raw material sources lead to significant variations in their impurity composition, crystal morphology, and solubility characteristics, resulting in distinct sulfate activation effects. Therefore, systematically investigating the activation patterns of diverse IWGs in ESSC is crucial for broadening gypsum sources and optimizing formulation design. Mechanical activation techniques, involving grinding and other mechanical forces, modify the microstructure of raw materials, increase specific surface area, and optimize particle size distribution, thereby enhancing material reactivity [19,20,21]. In recent years, this technology has achieved significant advancements in the activation of industrial solid waste [22]. Kenzhebek Akmalaiuly et al. [23] applied mechanical activation treatment to fly ash, which caused the hydration exothermic peak to occur earlier and improved the interface contact zone of the cement matrix, resulting in a compressive strength increase of up to 30%. Zhu et al. [24] demonstrated that the carbon sequestration capacity of coal gangue is significantly enhanced after mechanical activation. Wang et al. [25] used mechanical activation technology to pre-treat low-grade uranium–molybdenum ore, demonstrating its feasibility as an effective method for molybdenum recovery.

Mechanical activation techniques typically include both dry grinding and wet grinding methods. Compared to dry grinding, wet grinding offers significant advantages in activating solid waste. Jiao et al. [26] found in a comparative study that wet grinding more effectively promotes the formation of the sodium iodide phase. Guo et al. [27] demonstrated that wet grinding efficiently reduces and refines particle size within a shorter timeframe while optimizing particle morphology and distribution. In the preparation of cementitious materials, the presence of water during wet grinding lowers particle surface energy and minimizes agglomeration. Combined with the synergistic effects of chemical additives, this process produces finer particles with higher reactivity in treated solid waste, enabling it to better replace traditional cement [28,29,30,31]. Cementitious materials prepared by wet grinding exhibit outstanding early strength, optimized porosity, and enhanced durability, representing an effective technical approach to expanding the application scope of ESSC pastes.

In summary, although IWGs have been extensively studied for use in ESSC, systematic research on their activation characteristics and synergistic effects with wet grinding remains limited due to significant variations in the chemical composition of gypsum from different sources and types. Clarifying the influence of different gypsum sources on ESSC performance has important theoretical and practical implications for broadening the applicability of IWGs and optimizing ESSC formulation design.

Therefore, based on the above issues and research gaps, the core research objective of this study is to systematically clarify the activation characteristics from different source IWGs in ESSC and the synergistic enhancement mechanism of wet grinding technology and to provide theoretical and technical support for the large-scale application of IWG-based ESSC. Specifically, this study selected IWGs from different sources and combined them with granulated blast furnace slag (GGBS) and cement to prepare ESSC pastes. It systematically investigated the effects of gypsum type and wet grinding process on the performance of ESSC pastes. Moreover, X-ray diffraction (XRD), Fourier transform infrared spectroscopy (FTIR), thermogravimetric analysis (TG-DTG), and scanning electron microscopy (SEM) were used to characterize the hydration products and microstructure, further revealing the hydration mechanism of ESSC under the action of different IWGs and wet grinding. Finally, the feasibility of preparing high-performance ESSC cementitious materials using different IWGs was comprehensively evaluated, aiming to broaden the sources of gypsum raw materials for ESSC, optimize the formulation design and production process, and promote the green, low-carbon and large-scale development of the cement industry.

## 2. Materials and Methods

### 2.1. Raw Materials

**Figure 1:**
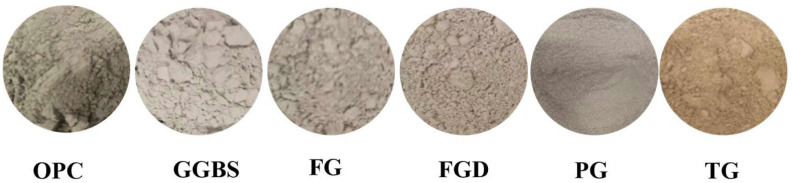
Raw materials used in the experiment.

**Figure 2:**
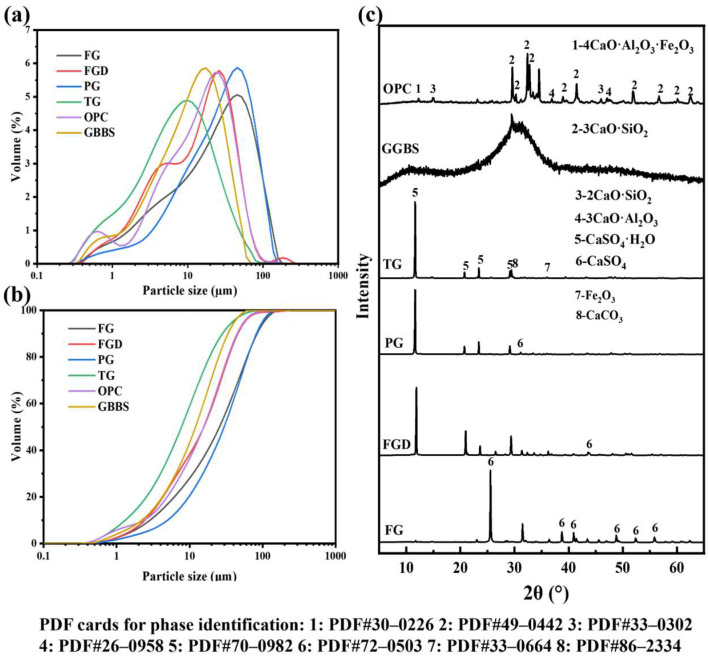
Particle size distribution patterns (**a**,**b**) and XRD patterns (**c**) of raw materials.

Figure 1 shows the images of the used raw materials (powders). The raw materials used in this study included ground granulated blast GGBS, OPC, and four types of IWGs: FG, FGD, PG, and TG. GGBS was grade S95 with an activity index ≥95% supplied by Shaoguan Iron & Steel Co., Ltd., Shaoguan, China; the OPC was P·O 52.5 cement produced by Huaxin Cement, Wuhan, China; FG originated from Laiyang, Shandong Province, China; FGD was sourced from Shandong Longkou Huadian Power Generation Co., Ltd., Yantai, China; PG was provided by Hubei Changyao New Materials Co., Ltd., Yichang, China; and TG was obtained from Longbai Sichuan Titanium Industry Co., Ltd., Mianzhu, China. Figure 2a,b show the particle size distribution curves of the raw materials, and Figure 2c shows the XRD patterns of the raw materials. Table 1 shows the chemical composition of the raw materials.

The primary chemical constituents of GGBS included CaO, SiO_2_, Al_2_O_3_, and MgO. Its XRD pattern exhibited no distinct diffraction peaks, indicating an amorphous state. The particle size ranged from 0.4 to 60 μm, with a D50 of 11.02 μm. The main chemical constituents of OPC included Al_2_O_3_, SiO_2_, SO_3_, and CaO, with a particle size range of 0.2 to 60 μm and a D50 of 11.64 μm. The primary phase of FGD, PG, and TG was CaSO_4_·2H_2_O. The primary phase of FG was CaSO_4_. FG and PG contained impurities such as phosphorus and fluorine, whereas TG contained significant amounts of impurities, including SiO_2_, Fe_2_O_3_, and TiO_2_, with a relatively low gypsum content. The particle size range for all four gypsums is 0.15 to 300 μm. The pH values of the four gypsums were as follows: FG (10.63) > TG (8.62) > FGD (7.41) > PG (4.24).

### 2.2. Sample Preparation and Experimental Procedures

**Figure 3:**
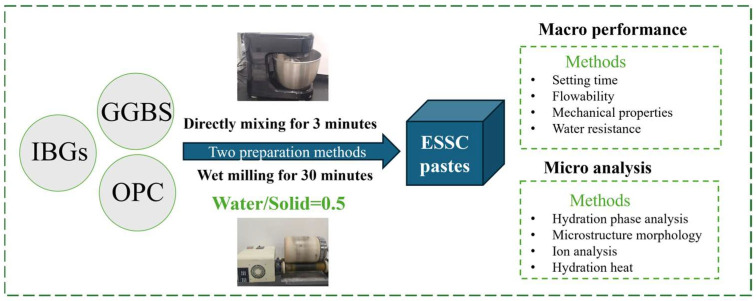
Process flow diagram of ESSC pastes preparation by direct mixing and wet grinding.

All raw materials were dried in an oven at 40 °C until reaching constant weight and then sieved through a 100-mesh screen. The resulting powder was used for the experiments. The solid raw materials were mixed at a dry mass ratio of 45:49:6 (IWGs:GGBS:OPC), and the water-to-solid ratio was kept at 0.5. Figure 3 shows the preparation method described in this study. In this study, two methods, namely direct mixing and wet grinding mixing, were adopted to prepare ESSC pastes. For direct mixing, the proportioned raw materials were fully mixed and added into a mixing bowl, followed by slow stirring for 120 s, a pause for 30 s, and rapid stirring for 60 s. The paste was then cast into 20 mm × 20 mm × 20 mm molds and vibrated repeatedly to remove air bubbles. After covering the mold surface with plastic wrap, the samples were cured in a standard curing room (20 ± 3 °C and 95% RH). After 3 d of hydration, the samples were demolded and further cured until the specified age. For wet grinding mixing, IWGs, GGBS, and OPC were mixed at a mass ratio of 45:49:6 and placed in a horizontal ceramic ball mill jar. The mass ratio of zirconia balls (6 cm:5 cm:3 cm:2 cm = 1:3:6:2) to powder was 6:1, and the water-to-solid ratio was set at 0.5. Powder and water were added to the ball milling jar. After ball milling for 30 min, the ESSC paste was immediately poured into the mold. The mold was vibrated, covered with plastic wrap, and the samples were demolded after standard curing for 3 d. Curing was continued until the specified age [32].

### 2.3. Experimental Test

The particle size distribution of the raw materials was measured using a Mastersizer 2000 laser particle size analyzer, Malvern Instruments Ltd., Malvern, UK. The raw materials were dispersed in analytical-grade anhydrous ethanol, and the measurement range was 0.02–2000 μm.

The chemical composition of the raw materials was quantitatively analyzed using a Zetium X-ray fluorescence spectrometer, Malvern Panalytical B.V., Almelo, The Netherlands.

The standard water content for consistency was tested for all samples using a Vicat apparatus, Wuxi Jianyi Instrument Machinery Co., Ltd., Wuxi, China according to GB/T 1346-2024 [33]. The samples were prepared at this standard water content, and both the initial and final setting times were measured for each sample.

The flowability of the pastes were determined according to GB/T 8077-2023 [34].

After curing for 3, 7, and 28 d, the compressive strength of all samples was tested using a TYE-3000, Wuxi Jianyi Instrument Machinery Co., Ltd., Wuxi, China press at a loading rate of 0.1 kN/s. The final compressive strength for each curing period was calculated as the average of three samples.

The early hydration heat release behavior of freshly mixed pastes was measured using an 8-channel isothermal calorimeter (TAM Air, TA Instruments, New Castle, DE, USA). Freshly prepared pastes were uniformly filled into glass ampoules, with a separate ampoule containing deionized water of equivalent specific heat capacity serving as the reference. The test duration was 144 h.

The softening coefficient and water absorption rate were used to characterize the water resistance of cementitious materials. The softening coefficient is defined as the ratio of wet compressive strength to dry compressive strength. For wet compressive strength, samples cured for 28 d were immersed in water for 48 h, and the water absorption rate before and after immersion was measured. For dry compressive strength, samples were dried in a 40 °C oven until constant weight was achieved. The softening coefficient (K) and water absorption rate (W) were calculated using Equations (1) and (2), respectively.

(1) $$K=\frac{K_{a}}{K_{b}}$$

(2) $$W=\frac{m_{1}-m_{0}}{m_{0}}$$

K_a_ and K_b_ represent the wet and dry compressive strengths of the sample, respectively (MPa). m_1_ and m_0_ denote the weight of the sample after water absorption and the weight of the sample before water absorption, respectively (g).

The ion dissolution of gypsum and freshly mixed pastes before and after wet grinding was measured using an inductively coupled plasma spectrometer, ICP-OES, Plasma 3000, NCS Testing Technology Co., Ltd., Beijing, China. Fresh pastes were prepared according to the mix design in Section 2.2. After 30 min, the fresh pastes were immediately centrifuged, and the ionic concentrations of the liquid phase were measured using ICP-OES. Simultaneously, the pH of the pore solution was measured using a pH meter (Phs-3E, Shanghai, China) after 30 min of gypsum and water hydration.

The samples to be tested were dried in an oven at 40 °C, then ground in a mortar, and the fine powder obtained after passing through a 20 μm sieve was used for XRD testing. The measurement range was 5° to 65°, with a step size of 0.02°, a scan speed of 2°/min, a voltage of 40 kV, and a current of 15 mA.

The infrared spectrum of the sample (powder sample identical to that used for XRD analysis) was measured using a Nicolet 6700 Fourier Transform Infrared Spectrometer, Waltham, MA, USA to evaluate changes in functional groups within samples at different ages before and after wet grinding. The test powder was uniformly mixed with KBr powder at a mass ratio of 1:100 and pressed into transparent pellets. The test region ranged from 4000 cm^−1^ to 400 cm^−1^.

TG-DTG of powder samples (the same as those used for XRD analysis) was performed using a TA Instruments SDT Q600 simultaneous thermal analyzer, New Castle, DE, USA. The experiments were conducted under a nitrogen atmosphere at a heating rate of 10 °C/min, with a temperature range from 25 °C to 1000 °C.

Hydrated samples were immersed in isopropanol for 24 h and then dried in a vacuum oven to constant weight. SEM analysis was performed using a TESCAN MIRA LMS, Brno, Czech Republic (3 kV, SE2 secondary electron detector) to investigate the microstructure and morphology of the hydrated samples. Before testing, the samples were coated with platinum using a Quorum SC7620 sputter coater, East Sussex, UK for 45 s.

The pore structure of ESSC paste after 28 d of hydration was measured using low-field nuclear magnetic resonance, Shanghai, China. Cubic samples measuring 20 mm × 20 mm × 20 mm were prepared.

## 3. Results and Discussions

### 3.1. Properties of Gypsums and Fresh Pastes

#### 3.1.1. Gypsum Dissolution Behavior Analysis

**Figure 4:**
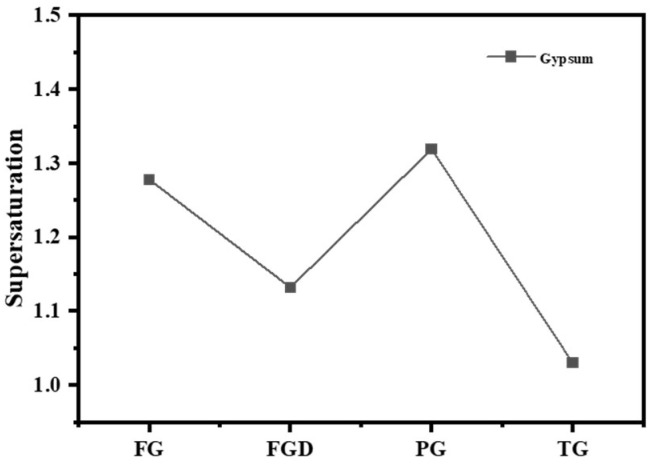
Supersaturation of different gypsum types.

Table 2 shows the dissolution rates of Ca^2+^ and SO_4_^2−^ ions and pH values in water for different IWGs. PG exhibited the highest concentration of Ca^2+^ (627.76 mg/L) and concentration of SO_4_^2−^ (1498.38 mg/L), indicating the highest solubility of PG in water. The solubility order of the four waste gypsums was PG > FG > FGD > TG. Gypsum supersaturation is typically defined as the square root of the ratio between the product of Ca^2+^ and SO_4_^2−^ concentrations in the liquid phase and the equilibrium product at CaSO_4_·2H_2_O dissolution (at 25 °C, Ksp = 1.41 × 10^−4^). Figure 4 shows the supersaturation of IWGs. All gypsum samples exhibited supersaturation values greater than 1, indicating that at room temperature, the selected IWGs demonstrated superior solubility in water compared to CaSO_4_·2H_2_O. Notably, FG and PG exhibited significantly higher supersaturation than FGD and TG, demonstrating superior sulfate activity.

#### 3.1.2. Setting Time

**Figure 5:**
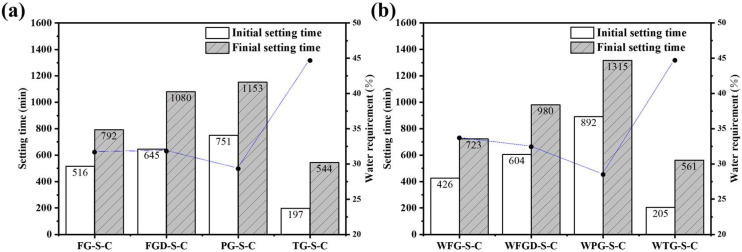
Setting time of the pastes (**a**) before and (**b**) after wet grinding.

Figure 5 shows the setting time of ESSC pastes before and after wet grinding. The setting rates of the four gypsum-containing pastes were TG > FG > FGD > PG. The paste containing TG exhibited an initial setting time of only 197 min and a final setting time of 544 min. For the other three samples, the setting times increased as the gypsum alkalinity decreased. The paste containing PG reached a final setting time of 1153 min, significantly longer than that of OPC, rendering it impractical for real-world applications. After wet grinding, the initial and final setting times decreased for the alkaline gypsum-containing WFG-S-C and WFGD-S-C samples. Conversely, the final setting time of the acidic PG-containing WPG-S-C sample increased by nearly 3 h, reaching 1315 min.

Compared to OPC, ESSC exhibited a longer setting time. This was attributed to insufficient early-stage alkalinity in the paste, which inhibited the initial dissolution and activation of GGBS. Additionally, soluble impurities such as phosphorus and fluorine in the gypsum hindered the formation of early-stage hydration products [35,36,37]. The pH levels of different IWGs varied significantly. At lower pH values, the dissolution and hydration of GGBS were inhibited. Furthermore, under acidic conditions, soluble impurities in the gypsum leached out more readily. Consequently, the setting time of pastes containing acidic PG increased markedly. Conversely, alkaline gypsum enhanced the overall alkalinity of the pastes while mitigating the effects of impurity ions. This improved the chemical environment and promoted early-stage hydration of the pastes [38]. It is noteworthy that TG exhibited weaker alkalinity than FG, yet possessed a significantly shorter setting time. This phenomenon was attributed to the substantial Fe_2_O_3_ content in TG. Iron-rich precursors inherently exhibit rapid setting properties, which explains the markedly accelerated setting time of TG-S-C [39].

#### 3.1.3. Flowability

**Figure 6:**
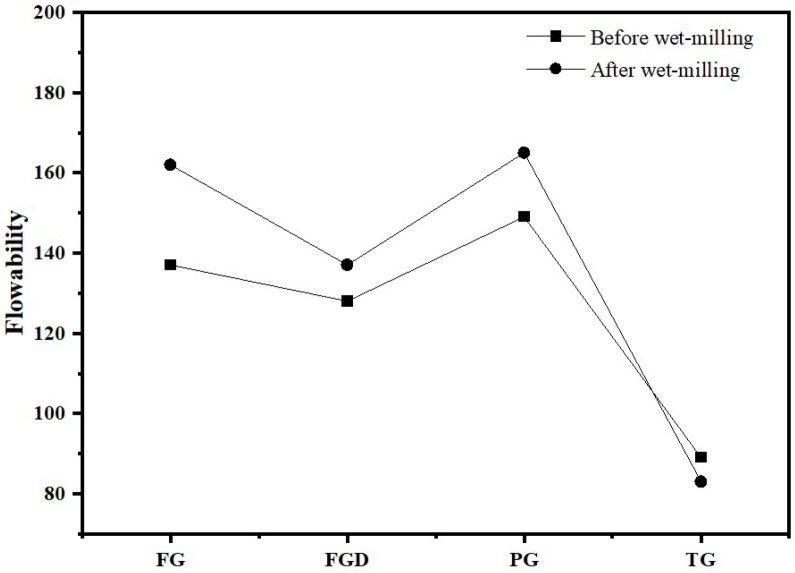
Flowability of ESSC pastes before and after wet grinding.

Figure 6 shows the flowability of ESSC pastes before and after wet grinding. As seen in the figure, before wet grinding, the flowability of the four samples ranked as follows: PG > FG > FGD > TG. Among them, the paste containing TG exhibited the poorest flowability, measuring only 89 mm. After wet grinding, the flowability of the TG-containing paste decreased to 83 mm, while the flowability of the other three gypsum pastes improved. FG and PG exhibited high solubility, resulting in higher flowability. In contrast, TG had a slow dissolution rate and contained a large amount of inert impurities, leading to significant interparticle friction and consequently poorer flowability.

### 3.2. Compressive Strength

**Figure 7:**
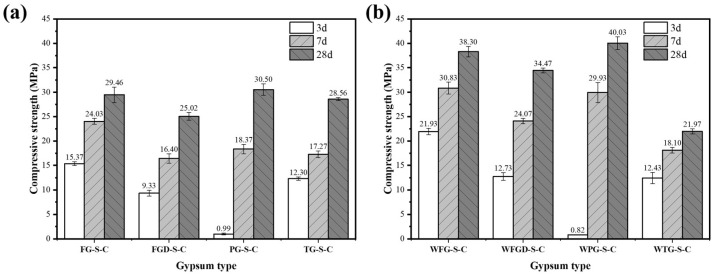
Compressive strength of pastes (**a**) before and (**b**) after wet grinding.

Figure 7 shows the compressive strength of ESSC pastes after 3, 7, and 28 d hydration before and after wet grinding. Samples containing different IWGs exhibited significant strength variations at different curing stages, with the wet grinding process demonstrating a marked improvement effect on ESSC pastes. Specifically, the PG-S-C sample exhibited a compressive strength of only 0.99 MPa at 3 d, while the FG-S-C, FGD-S-C, and TG-S-C samples recorded strengths of 15.37 MPa, 9.33 MPa, and 12.3 MPa, respectively, at the same age. As hydration progressed, all samples demonstrated significant increases in compressive strength at both 7 and 28 d. The strengths of WFG-S-C, WFGD-S-C, and WPG-S-C samples after 28 d of wet grinding hydration were 38.30 MPa, 34.47 MPa, and 40.03 MPa, representing increases of 30.01%, 37.77%, and 31.25%, respectively. In contrast, the strength of the TG-containing sample decreased to 21.97 MPa after 28 d of wet grinding hydration, a reduction of 23.07%.

The alkaline components of gypsum significantly influenced the 3 d strength of ESSC paste. The sample containing PG exhibited lower compressive strength after 3 d of hydration, a phenomenon linked to its acidic nature. The acidity of PG not only promoted the release of more soluble impurities but also neutralized OH^−^ ions in the system. This inhibited the dissolution and reaction of active slag ions, delaying the early hydration process of the paste and ultimately resulting in reduced early strength [38]. In contrast, the remaining three gypsum types exhibited alkaline pH values. The additional OH^−^ ions they provided more effectively stimulated the activity of GGBS, promoting the formation of substantial amounts of ettringite during the early hydration stage. This enabled the samples to achieve considerable compressive strength as early as 3 d of age. Wet grinding further accelerated the dissolution of slag, resulting in higher early strength. It should be noted that the three samples containing alkaline gypsum exhibited lower 28 d strength than the PG sample, suggesting that excessive alkaline components might hinder the sustained growth of ESSC pastes in later stages [16]. After wet grinding, the sample strength showed a significant improvement, indicating that wet grinding could enhance the performance of cementitious materials. The wet grinding process accelerated the dissolution of gypsum and GGBS, thereby improving the performance of ESSC cementitious materials.

### 3.3. Hydration Heat

**Figure 8:**
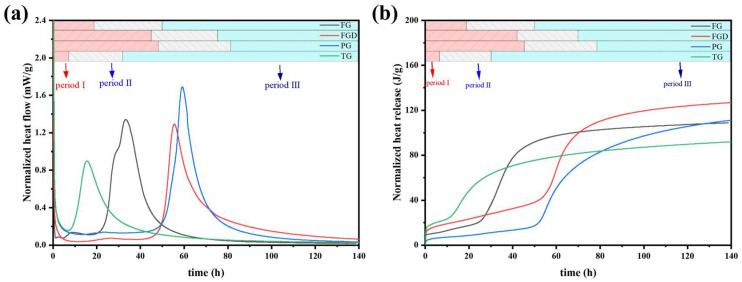
(**a**) Heat evolution and (**b**) cumulative heat release of the pastes.

Figure 8 shows the curves of hydration heat release and total hydration heat release for freshly mixed ESSC pastes. The hydration heat release trend of ESSC was similar to that of OPC, typically comprising three hydration stages: (1) the initial hydration and induction period, (2) the acceleration period, and (3) the deceleration and stabilization period [14]. The pastes containing different gypsum types exhibited markedly different heat release rates and heat release quantities.

Table 3 presents the end times of the induction period, main exothermic peaks, and cumulative heat release for all samples derived from the exothermic curves. Upon contact with water, the hydration exothermic heat immediately increased and persisted for 30 min. The TG-S-C paste completed its induction period at 6.05 h and exhibited a main exothermic peak, indicating that setting-promoting impurities such as Fe_2_O_3_ in TG significantly influenced the early hydration of the paste. Although the paste hydrated rapidly, its total heat release was relatively low at only 92.320 J/g, suggesting that excessively rapid setting might adversely affect subsequent hydration. Compared to the FGD-S-C and PG-S-C pastes, the heat release peak of the FG-S-C paste occurred significantly earlier. This demonstrates that high-alkalinity gypsum promoted early hydration in ESSC pastes and accelerated the dissolution of GGBS [14]. The main exothermic peaks of the FGD-S-C and PG-S-C pastes occurred at 55.81 h and 59.17 h, respectively, with slower early hydration reactions. Particularly in the PG-S-C paste, the acidic PG component exhibited stronger acidity, resulting in a longer induction period. Compared to FGD-S-C and PG-S-C, the TG-S-C and FG-S-C pastes exhibited broader peak shapes. In ESSC pastes, the main hydration exothermic peak corresponded to the extensive formation of C-(A)-S-H gel. The exothermic peak for pastes containing alkaline gypsum were broader, indicating that gypsum alkalinity significantly promotes the early formation of C-(A)-S-H gel.

### 3.4. Softening Coefficient and Water Absorption Rate

**Figure 9:**
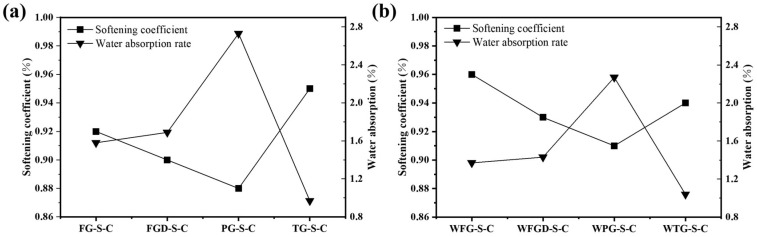
Softening coefficient and water absorption rate of pastes (**a**) before and (**b**) after wet grinding.

Figure 9 shows the softening coefficient and water absorption rate of each sample at 28 d of hydration before and after wet grinding. Samples containing alkaline gypsum exhibited higher softening coefficients and lower water absorption rates. The suitable alkaline environment promotes increased early-stage hydration products in the ESSC pastes, resulting in a denser sample surface and superior water resistance [20]. Although the PG-containing paste exhibited higher compressive strength after 28 d of hydration, the delayed initial hydration reaction resulted in a loose structure of surface hydration products. This also led to poor water resistance, indicating that the early alkalinity of ESSC pastes significantly impacts sample water resistance. Regulating the early alkalinity of ESSC pastes might thus be an effective approach to enhance their water resistance. During wet grinding, paste particles were ground in water to achieve finer and more uniformly distributed particles. These fine particles increased the specific surface area, not only accelerating the hydration reaction rate but also promoting more complete filling of pores by ESSC paste hydration products. This reduced interconnected pores and large-aperture defects within the material, thereby minimizing water penetration pathways. This was also the reason why the water resistance of the samples improved after wet grinding [29]. In summary, the softening coefficient and water absorption rate were key indicators of the water resistance of ESSC pastes. Both early-stage alkalinity control and wet grinding processes could enhance the softening coefficient by optimizing the hydration process and improving pore structure, thereby strengthening the material’s water resistance and durability. This provides crucial theoretical and practical support for subsequent performance optimization of ESSC pastes.

### 3.5. Hydration Phase Analysis

#### 3.5.1. XRD

**Figure 10:**
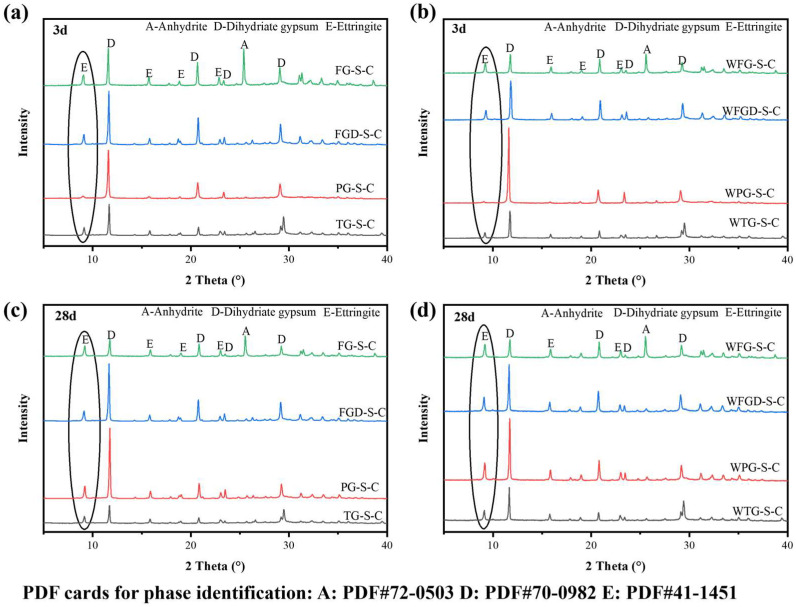
XRD patterns of hydrated pastes: (**a**) before wet grinding at 3 d; (**b**) after wet grinding at 3 d; (**c**) before wet grinding at 28 d; (**d**) after wet grinding at 28 d.

Figure 10 shows the XRD patterns of ESSC paste at 3 and 28 d after wet grinding. The primary hydration product in all samples was ettringite, alongside characteristic peaks of unhydrated anhydrous gypsum and dihydrate gypsum [40]. All samples exhibited characteristic diffraction peaks for ettringite and gypsum, with no other hydration products observed. Since C-(A)-S-H gel predominantly exists in an amorphous form, its characteristic diffraction peaks were difficult to discern in the figure. The characteristic peaks of ettringite could roughly indicate the amount of hydration products in the paste. The gypsum diffraction peaks correlate with the quantity of unhydrated IWGs. Despite identical phase compositions among the samples, the intensities of the characteristic diffraction peaks for hydration products varied, indicating differing hydration reaction processes [10].

After hydration 3 d, the characteristic peaks of ettringite and dihydrate gypsum were most pronounced at 9.1° and 11.6° in the samples. The FG-S-C sample exhibited a distinct characteristic peak for anhydrite at 25.4°. At 3 d of hydration, the hydration products primarily consisted of aluminate cement and dihydrate gypsum. The characteristic diffraction peaks of ettringite were most pronounced in FG-S-C, followed by FGD-S-C and TG-S-C, and were least evident in PG-S-C, consistent with strength development. After 28 d of hydration, the characteristic diffraction peaks of ettringite were enhanced in all samples, with the most pronounced increase observed in PG-S-C. Following wet grinding, the samples maintained the same pattern at 3 d and 28 d. However, wet grinding significantly amplified the ettringite diffraction peaks while further weakening the gypsum peaks, indicating that wet grinding accelerated paste hydration, leading to increased ettringite formation within the system.

#### 3.5.2. FTIR Results

**Figure 11:**
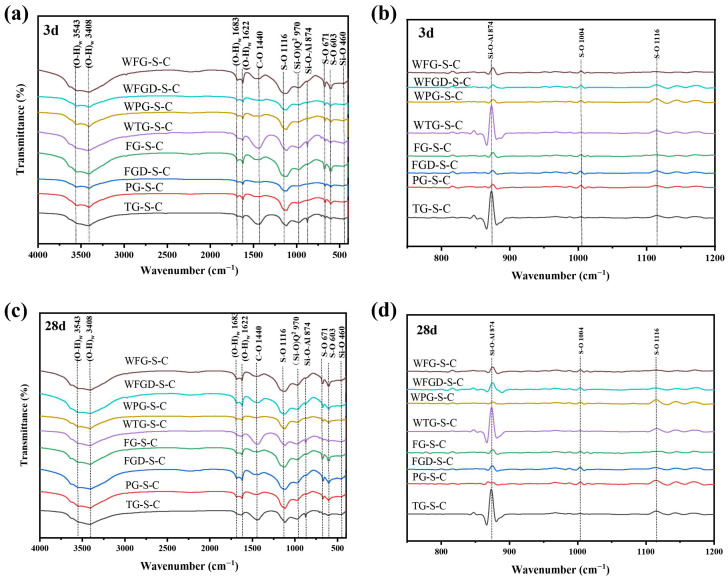
(**a**) FTIR results of pastes at 3 d; (**b**) the second derivative graph at 3 d; (**c**) FTIR results of pastes at 28 d; (**d**) the second derivative graph at 28 d.

Figure 11 shows the infrared spectra and the second derivative treatment of each sample at 3 and 28 d before and after wet grinding. The peaks at 3543 cm^−1^ and 3408 cm^−1^ could be attributed to O-H bond bending vibrations, while the peak at 1622 cm^−1^ was associated with both O-H bond bending and O-H bond stretching vibrations [16]. The S-O bond vibration peaks occurred at 1116 cm^−1^, 603 cm^−1^, and 671 cm^−1^, with the peaks at 603 cm^−1^ and 671 cm^−1^ associated with the bending vibration of SO_4_^2−^ [32]. The peak at 1116 cm^−1^ corresponded to the asymmetric stretching mode of SO_4_^2−^ ions, and these peaks were associated with calcite and unreacted gypsum [16]. The peak at 460 cm^−1^ corresponded to the Si-O bending vibration of GGBS, while the peak at 874 cm^−1^ observed in the C-(A)-S-H gel was attributed to the asymmetric stretching vibration of Al-O-Si.

At 3 d of hydration, FG-S-C exhibited the highest absorption peak intensity at the O-H and S-O positions, indicating increased formation of ettringite. This was followed by FG-S-C and FGD-S-C, consistent with XRD patterns. A distinct sharp peak at 874 cm^−1^ was observed in the TG-S-C sample, indicating the formation of a substantial amount of C-(A)-S-H gel with high polymerization and crosslinking degrees. At 28 d, no new vibrational peaks were observed in the infrared spectrum compared to the 3 d sample. A distinct peak at 1400–1450 cm^−1^ was observed in the TG-S-C sample, characteristic of the C-O stretching vibration in carbonates. This confirms the presence of calcite in the TG sample. After wet grinding, the peak corresponding to the C-(A)-S-H characteristic spectrum at 874 cm^−1^ was enhanced in all samples, indicating increased formation of C-(A)-S-H gel following wet grinding.

#### 3.5.3. Thermogravimetric Analysis 

**Figure 12:**
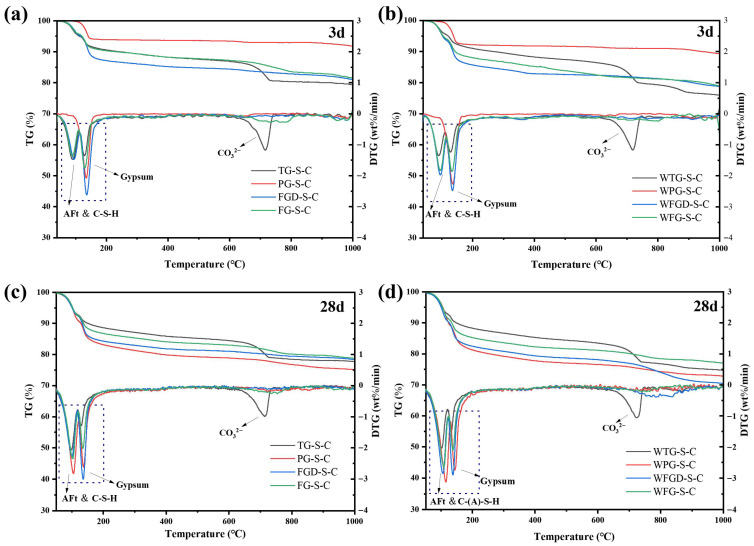
TG-DTG curves of pastes (**a**) before wet grinding at 3 d; (**b**) after wet grinding at 3 d; (**c**) before wet grinding at 28 d; (**d**) after wet grinding at 28 d.

Figure 12 shows the TG-DTG curves of each sample after 3 d and 28 d of hydration before and after wet grinding. Table 4 presents the mass loss of the samples across different temperature ranges. All pastes exhibited two distinct peaks within the 50–160 °C temperature range. These peaks were divided into two temperature intervals: 55–120 °C and 120–160 °C. The peak in the 55–120 °C range corresponded to the weight loss peak of C-(A)-S-H gel and ettringite, indicating the degree of sample hydration. The peak between 120 and 160 °C represented the weight loss peak of gypsum dehydration. The peak at 120–160 °C represented gypsum dehydration, reflecting gypsum consumption during hydration [41,42].

At 3 d of hydration, the weight loss of the samples in the 55–120 °C range followed the order: TG-S-C > FGD-S-C > FG-S-C > PG-S-C. The strong acidity of PG resulted in fewer early hydration products. After wet grinding, the peaks for all four samples in this temperature range increased. Within the 120–160 °C range, distinct dehydration peaks were observed across the four gypsum types, reflecting variations in gypsum content within the IWGs. Subsequently, all samples entered a stable dehydration zone between 160 and 600 °C, exhibiting no significant weight loss peaks [43]. Notably, within the 650–750 °C temperature range, TG-S-C exhibited a distinct weight loss peak, which was attributed to carbonate impurities present in TG. After 28 d of hydration, the first peak became more pronounced across all samples, accompanied by greater mass loss, indicating increased formation of hydration products. Following wet grinding, the peaks at 55–120 °C became more pronounced in the three samples other than WTG-S-C, with a significant increase in mass loss compared to pre-grinding. This indicated that wet grinding generated more hydration products, consistent with the subsequent compressive strength development.

### 3.6. Microstructure

#### 3.6.1. SEM Analysis

**Figure 13:**
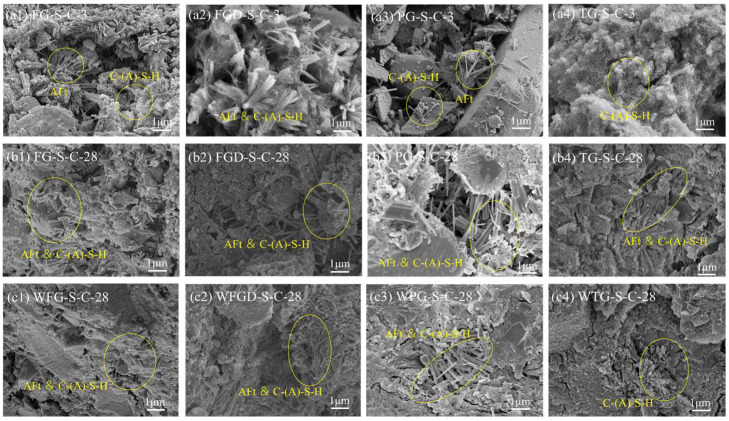
Microstructural morphology of pastes (**a1**–**a4**) before wet grinding at 3 d; (**b1**–**b4**) before wet grinding at 28 d; (**c1**–**c4**) after wet grinding at 28 d.

Figure 13 shows the microstructural morphology of each sample before and after wet grinding. The images revealed that after 3 d of hydration, the primary hydration products in the samples were ettringite and C-(A)-S-H gel. The TG-S-C sample exhibited a higher proportion of cross-linked C-(A)-S-H gel structures, while the FG-S-C sample contained more elongated ettringite crystals and a greater amount of granular C-(A)-S-H gel. The PG-S-C sample contained only a small amount of needle-like ettringite, with a significant amount of unreacted gypsum present. After 28 d of hydration, as GGBS continued to hydrate, the hydrated samples produced more hydration products and developed a denser structure. This is also why ESSC pastes exhibited higher late-stage strength [44,45]. After wet grinding, except for WTG-S-C, the samples exhibited a more compact structure, indicating that wet grinding accelerated mineral hydration and promoted the formation of additional C-(A)-S-H gel. The increased gel phase from hydration products resulted in a denser structure of the hardened pastes.

#### 3.6.2. Pore Structure

**Figure 14:**
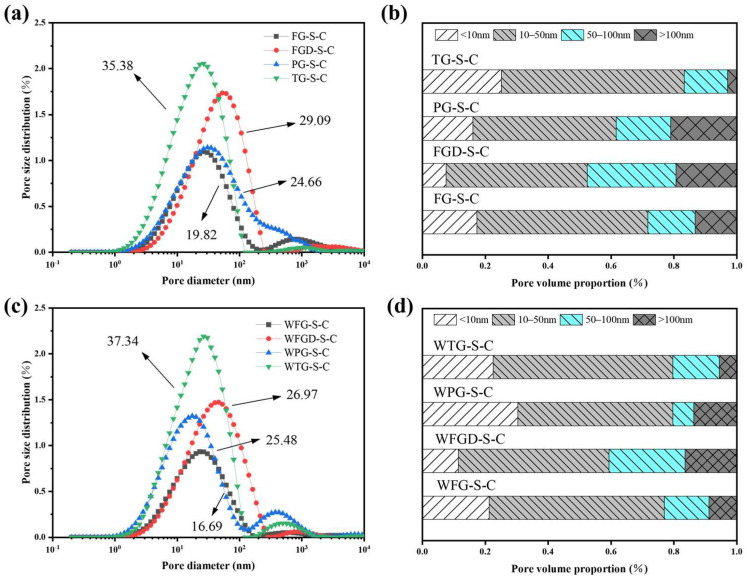
(**a**) Pore size distribution before wet grinding; (**b**) pore volume proportion before wet grinding at 28 d; (**c**) pore size distribution after wet grinding; (**d**) pore volume proportion after wet grinding at 28 d.

The pore structure of the sample directly influences its mechanical properties. Figure 14a,c display the pore size distribution curves of the ESSC samples after 28 d of hydration. The pore sizes in the cement sample could be categorized as follows: <10, 10–50, 50–100, and 100–5000 nanometers, corresponding to fine-, medium-, and large-sized gel pores and capillary pores in the cement samples, respectively [46]. Figure 14b,d show the distribution of pore sizes in ESSCs. The figures reveal that the porosity of the four samples follows the order TG-S-C > FGD-S-C > PG-S-C > FG-S-C. Before wet grinding, the TG-S-C sample contained the highest number of gel pores, indicating that hydration generated more C-(A)-S-H gel, consistent with FITR analysis. FGD-S-C exhibited the fewest gel pores and larger pore sizes, leading to its lower compressive strength in later stages. After wet grinding, the porosity of FG-S-C and FGD-S-C samples decreased, while the number of gel pores in WPG-S-C samples increased significantly, and the number of macropores decreased markedly. This indicated that wet grinding promoted sample hydration, resulting in the formation of more hydration products in the later stages, consistent with strength development.

### 3.7. Further Discussion

#### 3.7.1. Paste Dissolution Behavior Analysis

Table 5 shows the Ca^2+^ and SO_4_^2−^ ion concentrations and pH values in the pore solution of the ESSC pastes after 30 min of hydration for each sample before and after wet grinding. Before wet grinding, gypsum dissolved slowly, resulting in a low release rate of SO_4_^2−^ ions. This led to slow and insufficient formation of ettringite. The GGBS exhibited low activation efficiency, consequently yielding typically low early strengths that developed slowly.

**Figure 15:**
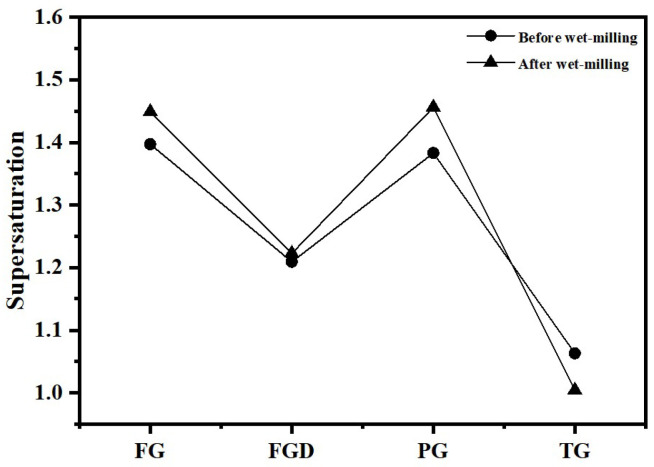
Supersaturation of gypsum in pastes before and after wet grinding.

Figure 15 displays the liquid-phase ion concentrations and calculated supersaturation results for each hydrated sample before and after wet grinding. After wet grinding, the SO_4_^2−^ ion concentration in the pore solution increased for all samples except TG, and the supersaturation of gypsum in the hydrated samples also increased. Higher supersaturation implied that more SO_4_^2−^ ions available to trigger GGBS reactions. This led to earlier, faster, and greater formation of ettringite. Additionally, nucleation rates accelerated, producing smaller crystal nuclei that interlock early on to form a robust skeletal structure. This resulted in a denser, more uniform microstructure, enhancing the early strength of ESSC pastes. Following wet grinding, the Ca^2+^ ion concentration in the cement paste decreased. Due to the combined effects of dissolution and pozzolanic reactions, wet grinding released more Ca^2+^ ions while simultaneously consuming Ca^2+^ ions to produce additional hydration products. Evidently, the rate of Ca^2+^ consumption exceeded that of Ca^2+^ production via hydrolysis, resulting in the observed decrease in Ca^2+^ concentration. Conversely, the SO_4_^2−^ concentration markedly increased after wet grinding, linked to the formation of ettringite. The rate of ettringite formation depended not only on SO_4_^2−^ concentration but more critically on the rate of AlO_2_^−^ released from slag hydration. The hydrolysis of GGBS glass and the breaking of Al-O bonds were inherently relatively slow processes. The relatively excess SO_4_^2−^ ions exerted a better stimulating effect on the GGBS. Once new AlO_2_^−^ ions were released through slag hydration, they could rapidly react with SO_4_^2−^ to form ettringite, continuously stimulating the deep hydration of the GGBS while also enhancing the later strength of the pastes. However, excessive SO_4_^2−^ ions can lead to excessive growth of ettringite, which has a high water content and loose crystal structure; the agglomeration and accumulation of excessive ettringite in the paste pores increase internal porosity, destroy the compactness of the microstructure, hinder the continuous formation of stratlingite, and ultimately have an adverse impact on the later strength. The optimal content of SO_4_^2−^ ions and the control method of ettringite growth to balance the positive and negative effects will be further explored in subsequent studies.

#### 3.7.2. Hydration Mechanism of Wet Grinding in Regulating Different ESSC

**Figure 16:**
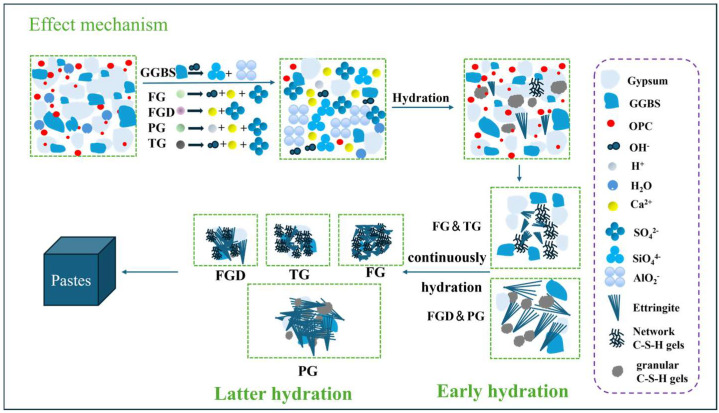
Activation mechanism of different gypsum types in ESSC pastes.

The activation mechanisms of different gypsum types on ESSC pastes are illustrated in Figure 16. During early hydration reactions, gypsum rapidly dissolved to produce Ca^2+^ and SO_4_^2−^ ions. At this stage, SO_4_^2−^ ions were abundant, and GGBS hydrolysis primarily relied on OH- ions generated from cement dissolution. These OH^−^ ions attacked the surface of GGBS particles, disrupting their Si-O and Al-O bonds. This released active ions such as Ca^2+^, Si^4+^, and Al^3+^ from GGBS into the solution. These ions then rapidly reacted with SO_4_^2−^ ions produced by gypsum dissolution to form ettringite and C-(A)-S-H gel. The highly alkaline gypsum generated more OH^−^ ions in the early-stage pastes, rapidly reacting to form a substantial cross-linked network of C-(A)-S-H gel.

Ca^2+^ + Al(OH)_4_^−^ + SO_4_^2−^ + OH^−^ + H_2_O → Ettringite (3)

Ca^2+^ + [SiO_4_]^4−^ + H_2_O → C-S-H gel (4)

Ca^2+^ + OH^−^ + Al(OH)_4_^−^ + [SiO_4_]^4−^ → C-A-S-H gel (5)

As the hydration of the pastes progressed, particularly the hydration of OPC, the alkalinity within the system gradually increased and stabilized. However, the continuous formation of ettringite consumed a significant amount of SO_4_^2−^ ions. Gypsum, which dissolved more slowly, contributed fewer SO_4_^2−^ ions to the system. This might result in insufficient SO_4_^2−^ ions around some slag particles, preventing their effective activation and resulting in fewer later-stage hydration products and consequently slower strength development in the later stages of ESSC pastes. In contrast, highly soluble gypsum ensured a continuous supply of SO_4_^2−^ ions in the system, continuously activating the slag and generating more hydration products. This is also why gypsum with high solubility exhibited superior later-stage strength. Notably, wet grinding affects both early hydration rate and long-term phase stability of ESSC pastes prepared with different IWG types, which are closely related to the continuous supply of sulfate ions and the formation of stratlingite. By refining the particle sizes of IWGs and GGBS and increasing their specific surface area, wet grinding effectively promotes the continuous dissolution of IWGs, ensuring a stable long-term supply of SO_4_^2−^ ions. This not only provides a material basis for the sustained formation of ettringite and stratlingite but also avoids insufficient activation of GGBS due to sulfate ion exhaustion in the late hydration stage. Meanwhile, wet grinding optimizes the particle size distribution of the paste, reduces pore size and porosity, and alleviates the risks of delayed ettringite formation and sulfate attack, which is conducive to maintaining long-term microstructural stability.

## 4. Conclusions

This experiment investigated the effects of different IWGs and wet grinding on the mechanical properties, hydration process, and hydration products of ESSC pastes. Based on the research findings, the following conclusions can be drawn:

(1) Different types of IWGs did not alter the types of ESSC hydration products, which mainly included ettringite, C-(A)-S-H gel and stratlingite. However, they led to obvious differences in compressive strength: FG-S-C and TG-S-C had higher early compressive strength due to their higher initial pH values, while PG-S-C exhibited better late compressive strength, benefiting from the high solubility of PG that ensured a continuous sulfate supply for sustained hydration.
(2) Setting time and hydration heat analyses indicated that FGD-S-C and PG-S-C exhibited longer setting times and induction periods due to their lower initial pH values. Conversely, the higher initial pH of FG-S-C and TG-S-C accelerated the dissolution and hydration of GGBS.
(3) Microstructural analysis revealed that the quantity and volume of hydration products in ESSC gradually increased with curing time, thereby enhancing compressive strength. Wet grinding treatment increased gel pores and reduced capillary pores in the samples, optimizing the pore structure and improving compressive strength.
(4) Wet grinding technology reduced particle surface energy and agglomeration, enhancing the reactivity of ESSC and promoting paste strength development. Compared with existing literature on gypsum-based cementitious materials, the softening coefficient of ESSC prepared using FG in this study reached 0.96, significantly higher than the typically reported range of 0.60–0.85 for conventional industrial by-product gypsum-based cementitious materials. Additionally, the 28-day compressive strength of WPG-S-C reached 40.03 MPa, representing a 31.25% increase. This strength enhancement is notably more pronounced compared to most studies employing conventional grinding or single alkali activation methods.

In summary, this study provides a crucial theoretical foundation for the application of various IWGs in low-carbon cementitious materials, offering an effective technical approach to reducing carbon emissions in cement systems. However, this study only examined the effects of four types of IWGs on ESSC and did not investigate the system’s durability. The long-term phase stability and structural integrity of ESSC pastes containing IWGs warrant further investigation. The continuous sulfate release from different IWGs and the potential for delayed ettringite formation may compromise the material’s dimensional stability and microstructural integrity under prolonged humid conditions. The evaluation of the long-term phase stability of wet-ground ESSC pastes still needs further improvement. Future research should systematically study the long-term phase evolution of wet-ground ESSC pastes under different service conditions, clarify the regulatory role of wet grinding in the long-term hydration mechanism, and provide a more comprehensive theoretical basis for improving the long-term durability of ESSC materials.

## Figures and Tables

**Table 1:** Chemical compositions of raw materials.

Materials	CaO	Al_2_O_3_	SiO_2_	SO_3_	Fe_2_O_3_	MgO	Other	LOI	pH
OPC	63.30	4.98	19.17	3.85	3.29	1.25	2.52	1.64	12.52
GGBS	42.83	14.43	30.21	2.33	0.30	7.96	1.94	N.D	9.73
FG	39.30	0.28	0.48	54.45	0.56	0.34	2.32	2.27	10.63
FGD	32.20	0.60	1.51	42.98	0.20	0.51	0.36	21.64	7.41
PG	31.93	0.27	1.80	41.87	0.08	0.04	2.03	21.98	4.24
TG	33.66	0.93	7.43	20.88	6.59	1.32	4.20	24.99	8.62

**Table 2:** Ca^2+^ and SO_4_^2−^ concentrations and pH values in gypsum pore solution.

Sample Label	Ca^2+^ (mg/L)	S (SO_4_^2−^) (mg/L)	pH
FG	615.85	1434.35	10.63
FGD	550.37	1258.35	7.41
PG	627.76	1498.38	4.24
TG	499.31	1147.37	8.62

**Table 3:** Duration of the three hydration stages (h) and heat release (wt%).

Samples	Inflection (h)	Exothermic Rate (mW/g)	Peak (h)	Exothermic Rate (mW/g)	Total Heat Release (J/g)
FG-S-C	18.51	0.11	33.29	1.34	108.87
FGD-S-C	42.07	0.07	55.81	1.29	128.04
PG-S-C	45.40	0.16	59.17	1.70	111.88
TG-S-C	6.05	0.16	15.53	0.90	92.32

**Table 4:** Mass Loss (wt%) at different temperature ranges.

Samples	3 d		28 d	
	55–120 °C	120–160 °C	55–120 °C	120–160 °C
FG-S-C	4.03	4.72	7.28	4.57
FGD-S-C	4.65	7.01	7.42	6.87
PG-S-C	0.13	5.76	8.85	6.27
TG-S-C	4.74	3.61	6.96	3.05
WFG-S-C	5.69	5.05	8.92	5.04
WFGD-S-C	6.39	6.55	8.07	6.71
WPG-S-C	0.34	7.97	9.03	7.54
TG-S-C	4.42	3.44	6.67	3.45

**Table 5:** Ca^2+^ and SO_4_^2−^ concentrations and pH values in the borehole solution.

Sample Label	Ca^2+^ (mg/L)	S (SO_4_^2−^) (mg/L)	pH
FG-S-C	775.78	1359.35	12.54
FGD-S-C	637.91	1235.89	11.98
PG-S-C	712.64	1447.53	11.76
TG-S-C	513.79	1189.24	12.23
WFG-S-C	744.18	1524.24	11.76
WFGD-S-C	595.63	1355.37	11.23
WPG-S-C	689.24	1662.25	11.11
WTG-S-C	474.14	1149.74	11.57

## Data Availability

The original contributions presented in this study are included in the article. Further inquiries can be directed to the corresponding author.
